# The microphthalmia-associated transcription factor (*Mitf*) gene and its role in regulating eye function

**DOI:** 10.1038/s41598-019-51819-0

**Published:** 2019-10-28

**Authors:** Andrea García-Llorca, Snaefridur Gudmundsdottir Aspelund, Margret Helga Ogmundsdottir, Eiríkur Steingrimsson, Thor Eysteinsson

**Affiliations:** 1 Department of Physiology, Biomedical Center, Faculty of Medicine, University of Iceland, Vatnsmyrarvegur 16, 101 Reykjavík, Iceland; 2 Department of Ophthalmology, Landspitali National University Hospital, Eiriksgata 37, 101 Reykjavik, Iceland; 3 Department of Anatomy, Biomedical Center, Faculty of Medicine, University of Iceland, Sturlugata 8, Reykjavík, Iceland; 4 Department of Biochemistry and Molecular Biology, Biomedical Center, Faculty of Medicine, University of Iceland, Sturlugata 8, Reykjavík, Iceland

**Keywords:** Sensory processing, Molecular medicine

## Abstract

Mutations in the microphthalmia-associated transcription factor (*Mitf*) gene can cause retinal pigment epithelium (RPE) and retinal dysfunction and degeneration. We examined retinal and RPE structure and function in 3 month old mice homo- or heterozygous or compound heterozygous for different *Mitf* mutations (*Mitf*^*mi*-*vga9*/+^, *Mitf*^*mi*-*enu22*(*398*)^/*Mitf*^*mi*-*enu22*(*398*)^, *Mitf*^*Mi*-*Wh*/+^ and *Mitf*^*Mi*-*Wh*^/*Mitf*^*mi*^) which all have normal eye size with apparently normal eye pigmentation. Here we show that their vision and retinal structures are differentially affected. Hypopigmentation was evident in all the mutants while bright-field fundus images showed yellow spots with non-pigmented areas in the *Mitf*^*mi*-*vga9*/+^ mice. *Mitf*^*Mi*-*Wh*/+^ and *Mitf*^*Mi*-*Wh*^/*Mitf*^*mi*^ mice showed large non-pigmented areas. Fluorescent angiography (FA) of all mutants except *Mitf*^*mi*-*vga9*/+^ mice showed hyperfluorescent areas, whereas FA from both *Mitf*^-*Mi*-*Wh*/+^ and *Mitf*^*Mi*-*Wh*^/*Mitf*^*mi*^ mice showed reduced capillary network as well as hyperfluorescent areas. Electroretinogram (ERG) recordings show that *Mitf*^*Mi*-*Wh*/+^ and *Mitf*^*Mi*-*Wh*^/*Mitf*^*mi*^ mice are severely impaired functionally whereas the scotopic and photopic ERG responses of *Mitf*^*mi*-*vga9*/+^ and *Mitf*^*mi*-*enu22*(*398*)^/*Mitf*^*mi*-*enu22*(*398*)^ mice were not significantly different from wild type mice. Histological sections demonstrated that the outer retinal layers were absent from the *Mitf*^*Mi*-*Wh*/+^ and *Mitf*^*Mi*-*Wh*^/*Mitf*^*mi*^ blind mutants. Our results show that *Mitf* mutations affect eye function, even in the heterozygous condition and that the alleles studied can be arranged in an allelic series in this respect.

## Introduction

One of the major causes of visual impairment and blindness in humans are retinal degenerations. These are either due to inheritance of mutations in genes known to cause defects in the retina or the retinal pigment epithelium (RPE)^1–5^. Animal models with either inherited or induced degeneration are of proven value to understand the molecular basis of such degenerations and to assess the functional state of degenerating retina^6–8^. Many of the mutations known to cause retinal degeneration can be traced to retinal structural proteins and to proteins involved in phototransduction^1,8^. Other mutations causing retinal degenerations are related to dysfunctional proteins expressed in retinal neurons, the photoreceptors^7,9,10^, or RPE cells^4,6,11^. The most common mutations leading to retinal degenerations have been found in genes expressed in photoreceptors, while known mutations in genes specifically expressed in the RPE are fewer^8,11^. Hence, mouse models of retinal degenerations involving mutations in genes expressed specifically in the RPE are of interest, since they can shed light on the role that RPE dysfunction by itself play in such degenerations^4^. Mutations in RPE-specific genes known to cause retinal degenerations in humans include *RPE65* (which is also expressed in red/green cones in humans^12^), *LRAT*, and *MERTK*; mouse models are available for investigating all of them^6,11,13,14^.

Several transcription factors have an important role in determining the eye phenotype. The most studied of these transcription factors, are the homeodomain transcription factors *Crx*^15^ and *Otx2*^16–20^, the retina-specific basic motif-leucine zipper (bZIP) transcription factor NRL^21,22^, and the microphthalmia-associated transcription factor family *Mitf*-*Tfe*^15,18,23^. Mutations in these transcription factors can lead to retinal degeneration. NRL controls the expression of rod genes^22^, and at least 20 of these target genes are associated with human retinal dystrophies; the Nrl-KO mouse has no rods and no expression of rod-specific genes^24^. The MITF protein is a member of the MYC supergene family of basic-helix-loop-helix-leucine-zipper (bHLHZip) transcription factors and is known to regulate the expression of cell specific target genes by binding DNA as a homodimer or as heterodimer with related proteins. The MITF transcription factor is composed of a basic DNA binding domain, and helix-loop-helix and zipper regions, both important for dimerization^25–27^. The expression of these transcription factors in the eye differs, with the *CRX* and *NRL* genes expressed in photoreceptors^15,22^, whereas different isoforms of *MITF* are expressed preferentially or exclusively at birth in the retinal pigment epithelium (RPE)^26,28^, *OTX2* is expressed in both the RPE, bipolar cells and photoreceptors^16,17^. The *Mitf* gene is known to affect the morphology and function of the retina as assessed by histology and electroretinography^23,29^, although a limited number of mutant alleles have been examined. Mutations in other genes expressed solely in the RPE, such as *LRAT* and *MERTK*, have been identified in patients with early-onset retinal degeneration, and mice carrying knockout (KO) mutations of these genes have the same effect^6,8^. Interestingly, the *RPE65* gene is expressed in both mouse and human RPE and subpopulations of cone photoreceptors^12,30^, and mutations in the gene can lead to severe retinal degenerations affecting both rods and cones. However, available mouse models of retinal degeneration or dysfunction caused by known mutations in RPE-specific genes are relatively few, compared to those caused by mutations in photoreceptor genes^6,17,18^.

In mice, there are over 30 distinct alleles in the *Mitf* gene that have different phenotypic effects, and these can be arranged in allelic series according to their phenotypic severity, from normal to microphthalmic animals with osteopetrosis^31,32^. Common to all the *Mitf* mouse mutations are defective neural-crest-derived melanocytes, resulting in a reduction or lack of pigmentation in the coat, inner ear and eye^31^. *Mitf* mutant mice may have defects in some or all of the various tissues listed above, for instance, reduced eye size or microphthalmia^26,31,33^, early onset of deafness^34^, a failure of secondary bone resorption and loss of pigmentation^25,35^. In humans, *MITF* mutations have been found in patients with the pigmentation and deafness disorders Waardenburg (OMIM # 193510)^36,37^ and Tietz (OMIM # 103500)^38,39^ syndromes and recently, they have been associated with microphthalmia and osteopetrosis^40^. *Mitf* mutations affect both kinds of pigment cells in the eye, the neural-crest derived melanocytes and the neuroepithelial-derived RPE cells^41–43^. *Mitf* promotes RPE differentiation, regulates the proliferation of the RPE during development and consequently their retinal function^25,42,44,45^. There is evidence that the *Mitf*-*Tfe* family of transcription factors regulate the expression of vascular endothelial growth factor (VEGF) in the RPE^46^ and thus may affect retinal vascular function.

The purpose of this study was to examine the eye phenotype in mice with four different mutations in the *Mitf* gene that have mild or intermediate effects on eye development in heterozygous, homozygous or in compound heterozygous conditions. Our primary focus is on the structure and function of the retina and the RPE in mice with different mutations in a gene that is expressed in the RPE.

## Results

### Mice used in the study

**Figure 1:**
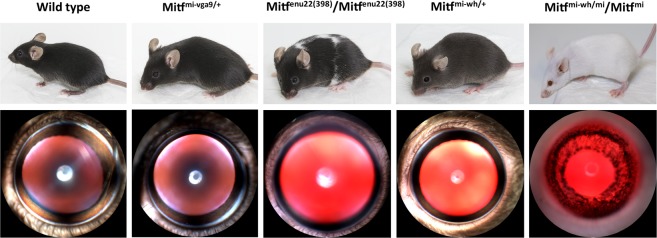
Phenotype of the mice used in this study. Coat appearance of mice examined in this study (upper panel). Representative images of the anterior part of the eye after pupil dilatation (n = 6) (lower panel). All eyes are of normal size. The overall appearance of the eyes of all mutants, except the *Mitf*^*mi*-*vga9*/+^ mouse, is lighter than that of the wild type.

The *Mitf* mutations studied here have all been described previously^32^ and are listed in Table 1. Heterozygotes carrying *Mitf*^*mi*-*vga9*/+^and *Mitf*^-*Mi*-*Wh*/+^ mutations have normal eye size: although the former has normal coat pigmentation, the latter have grey diluted coat colour (Fig. 1; Table 1)^32,47^. Mice homozygous for the *Mitf*^*mi*-*enu22*(*398*)^ mutation have a black coat with a white belly and unpigmented spots over the rest of the body, and dark ruby eyes of normal size. The *Mitf*^*Mi*-*Wh*^/*Mitf*^*mi*^ mice have a white coat whereas eye size is normal (Fig. 1). These particular mutations, in heterozygous, homozygous or compound heterozygous conditions, were selected for study as they all have apparently normal eye development. After application of the mydriatic tropicamide, the irises of all mutants fully dilate, except the *Mitf*^*Mi*-*Wh*^/*Mitf*^*mi*^ mice, which show an iris that is less pigmented than that of wild type mice, and which dilates only partially with tropicamide, possibly due to hypoplasia (Fig. 1).

**Table 1:** *Mitf* mutations used in this study. Adopted from Steingrimsson *et al*. ^32^ and Bauer *et al*.^47^.

Allele	Symbol	Heterozygote	Homozygote	Lesion
microphthalmia	*Mitf* ^*mi*^	Iris pigment less than in wild type; spots on belly, head and tail	White coat, eyes small and red; deficiency of mast cells, spinal ganglia, adrenal medulla and dermis smaller than normal; incisors fail to erupt, osteopetrosis; inner ear defects	3-bp deletion in basic domain
white	*Mitf* ^M^ ^*i*- *Wh*^	Coat color lighter than dilute (*d*/*d*); eyes dark ruby; spots on feet, tail and belly; inner ear defects	White coat; eyes small and slightly pigmented; spinal ganglia, adrenal medulla and dermis smaller than normal; inner ear defects; reduced fertility	I212N
enu-22(398)	*Mitf* ^*mi*- *enu22*( *398*)^	Normal	Black with white belly and spots over the rest of the body.	Q26STOP
VGA-9	*Mitf* ^*mi*- *vga9*^	Normal	White coat, eyes red and small; inner ear defects	transgene insertion and 882-bp deletion

### Varying hypopigmentation in *Mitf* mutant eyes

**Figure 2:**
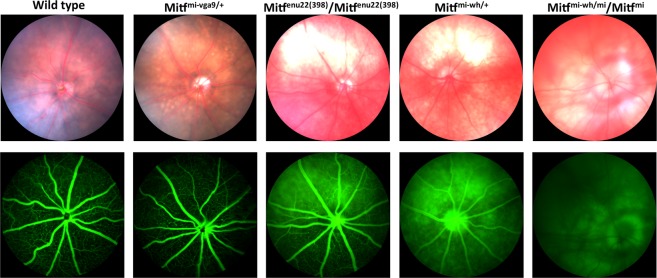
Fundus photographs from 3 month old mice. Bright field images of wild type and mutant mice (n = 6) (upper panel). Note the spots of depigmentation in the *Mitf*^*mi*-*vga9*/+^mouse bright field fundus image, and the hypopigmentation present in all mutants. Fluorescein angiography images obtained from wild type and mutant mice (n=6) (lower panel). Blurred fluorescent areas are seen in *Mitf*^*mi*-*enu22*(*398*)^/*Mitf*^*mi*-*enu22*(*398*)^ and *Mitf*^*Mi*-*Wh*/+^animals, possibly corresponding to the non-pigmented areas in the bright field images.

Representative bright field and fluorescent fundus images obtained with a rodent fundus camera from the eyes of wild type (n = 6) and mutant mice are shown in Fig. 2. As demonstrated by the bright field images (Fig. 2, upper panel) the optic disk areas were of normal size in all mutants, further indicating that the eyes of these mutant mice develop to a normal size in all cases. All the mutant mice show some hypopigmentation in their fundi, varying in degree. The fundi of the *Mitf*^*mi*-*vga9*/+^ mice (n = 6) show discrete yellow lesions, with only minor hypopigmentation overall, but discreet pigment mottling scattered throughout the entire fundus. The bright field fundus images from *Mitf*^*mi*-*enu22*(*398*)^/*Mitf*^*mi*-*enu22*(*398*)^ mice (n = 6) reveal large unpigmented lesions in both eyes, usually in the superior half of the fundus, with irregular borders. The fundi of the *Mitf*^-M*i*-*Wh*/+^ mice (n = 6) also show hypopigmentation and comparable large non-pigmented areas, although more extensive, but no pigment mottling. The fundus images of the *Mitf*^*Mi*-*Wh*^/*Mitf*^*mi*^ mice (n = 6) show more widespread lack of pigmentation and larger apparent RPE lesions, without any pigment mottling.

The fluorescent angiography (FA) images from wild type (n = 6) and *Mitf*^*mi*-*vga9*/+^ mice (n = 6) showed normal retinal vasculature with no hyperfluorescent signals, and extensive capillaries emanating from the major vessels (Fig. 2, lower panel). The FA of the *Mitf*^*mi*-*enu22*(*398*)^/*Mitf*^*mi*-*enu22*(*398*)^ mice (n = 6) showed hyperfluorescent areas where there are hypopigmented lesions in the superior half of the fundus, but with capillaries present. However, FA of the *Mitf*^*Mi*-*Wh*/+^ mice show hyperfluorescent regions across the entire fundus, and a clear reduction in the capillary network. *Mitf*^*Mi*-*Wh*^/*Mitf*^*mi*^ mice showed hardly any fluorescence in their retinal vasculature in FA, but extensive hyperfluorescent signals that appear to emanate from the choroidal vasculature over nearly the entire fundus. These hyperfluorescent signals correspond in area to the hypopigmented lesions evident in the bright field fundus images.

### *Mitf* has an impact on visual function

**Figure 3:**
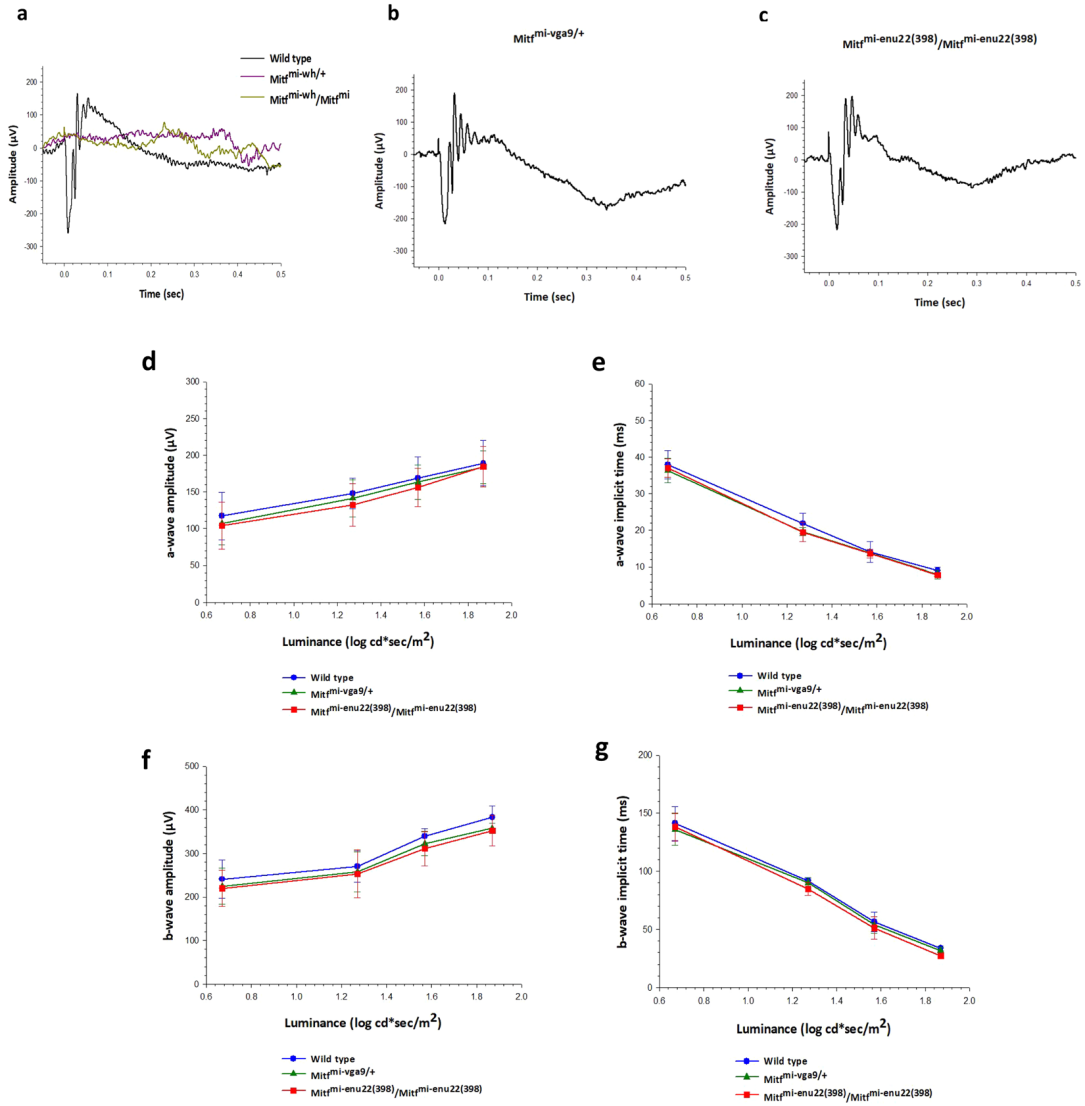
Dark adapted ERG recordings. (**a**–**c**) Representative ERG recordings from a wild type mouse (**a**, dark trace), and from mice with four different mutations in the *Mitf* gene, in response to a single flash light stimulus of 1.87 log cd s/m^2^. ERG responses recorded from *Mitf*^-*Mi*-*Wh*/+^and *Mitf*^*Mi*-*Wh*^/*Mitf*^*mi*^ mice could not be detected above baseline (**a**, coloured traces), while responses from *Mitf*^*mi*-*vga9*/+^ (**b**) and *Mitf*^*mi*-*enu22*(*398*)^/*Mitf*^*mi*-*enu22*(*398*)^ (**c**) showed a- and b-waves. One-way ANOVA followed by Bonferroni’s post-hoc comparisons test (wild type set as control) was used. Data are presented as means ± SEM, n = 6, P > 0.05.

ERG recordings were obtained from wild type and mutant mice. Dark-adapted ERG responses evoked by the 1.87 log cd sec/m^2^ stimulus are presented in Fig. 3a–c. Two of the *Mitf* mutant mice, *Mitf*^-*Mi*-W*h*/+^and *Mitf*^*Mi*-*Wh*^/*Mitf*^*mi*^ showed no detectable dark-adapted ERG responses above baseline (Fig. 3a), suggesting that they have severely impaired retinal function. The dark-adapted ERG responses of these mice were all less than could be detected above baseline regardless of stimulus luminance (data not shown), while the other two mutant mice (*Mitf*^*mi*-*vga9*/+^ and *Mitf*^*mi*-*enu22*(*398*)^/*Mitf*^*mi*-*enu22*(*398*)^) showed dark-adapted ERG a- and b-waves that were not significantly different in amplitude (Fig. 3d,f) or implicit time (Fig. 3e,g) from wild type at any of the levels of the stimulus luminance tested (P > 0.05). The mean amplitude of the dark-adapted a-wave of the ERG in response to the 1.87 log cd sec/m^2^ stimulus was 189.1 ± 30.9 µV in wild type (n = 6), 183.4 ± 22.4 µV in *Mitf*^*mi*-*vga9*/+^ mice (n = 6), and 184.4 ± 27.8 µV in *Mitf*^*mi*-*enu22*(*398*)^/*Mitf*^*mi*-*enu22*(*398*)^ mice (n = 6). The mean amplitude of the dark-adapted b-wave of the ERG in response to the same stimulus was 383.3 ± 26.0 µV in wild type mice, while it was 357.6 ± 11.5 µV in *Mitf*^*mi*-*vga9*/+^ mice (n = 6) and 352.4 ± 35.6 µV in *Mitf*^*mi*-*enu22*(*398*)^/*Mitf*^*mi*-*enu22*(*398*)^ mice (n = 6). The a- and b-wave implicit times were normal in those mutants that showed dark-adapted ERG responses at any of the levels of the stimulus luminance tested (P>0.05). The mean of the a-wave implicit time in response to the 1.87 log cd sec/m^2^ stimulus was 8.9 ± 0.8 µV in wild type, while it was 8.0 ± 1.1 µV in *Mitf*^*mi*-*vga9*/+^ mice, and 7.9 ± 0.5 µV in *Mitf*^*mi*-*enu22*(*398*)^/*Mitf*^*mi*-*enu22*(*398*)^ mice. The mean of the b-wave implicit time in response to the 1.87 log cd sec/m^2^ stimulus in normal mice was 33.7 ± 2.0 µV, whereas it was 31.8 ± 2.6 µV in *Mitf*^*mi*-*vga9*/+^ mice, and 27.1 ± 1.8 µV in *Mitf*^*mi*-*enu22*(*398*)^/*Mitf*^*mi*-*enu22*(*398*)^ mice.

**Figure 4:**
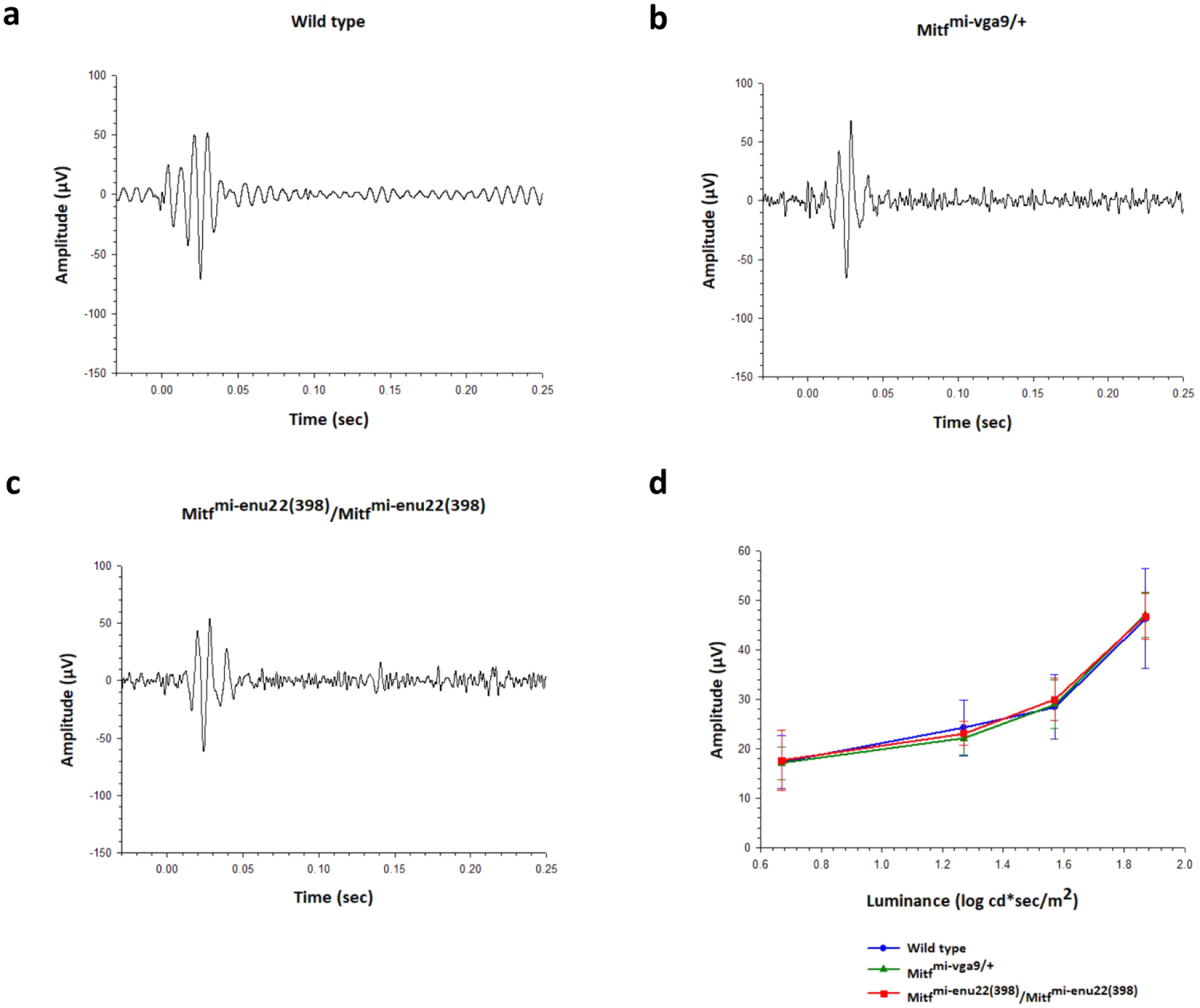
Dark-adapted OPs recordings. (**a**–**c**) Representative OPs recordings from wild type mouse (**a**, dark trace), and from mice carrying four different *Mitf* gene genotypes in response to a single flash light stimuli of 1.87 log cd s/m^2^. (**d**) The mean amplitudes of dark-adapted OPs. One-way ANOVA followed by Bonferroni’s post-hoc comparisons test (wild type set as control) was used. Data are presented as means ± SEM, n = 6, P > 0.05.

The dark-adapted oscillatory potentials, evoked by the 1.87 log cd sec/m^2^ stimulus but isolated with a 100–500 Hz digital filter are presented in Fig. 4a–c. These wavelets riding on the dark-adapted b-wave, reflecting inner retinal activity, were not significantly different between *Mitf*^*mi*-*vga9*/+^, *Mitf*^*mi*-*enu22*(*398*)^/*Mitf*^*mi*-*enu22*(*398*)^ and wild type mice at any of the levels of the stimulus luminance tested (P > 0.05) (Fig. 4d). The mean amplitude of the dark-adapted OPs in response to the 1.87 log cd sec/m^2^ stimulus was 43.4 ± 15.5 µV in normal mice, 39.0 ± 6.5 µV in *Mitf*^*mi*-*vga9*/+^ mice, and 44.1 ± 6.4 µV in *Mitf*^*mi*-*enu22*(*398*)^/*Mitf*^*mi*-*enu22*(*398*)^ mice in response to the same stimulus.

**Figure 5:**
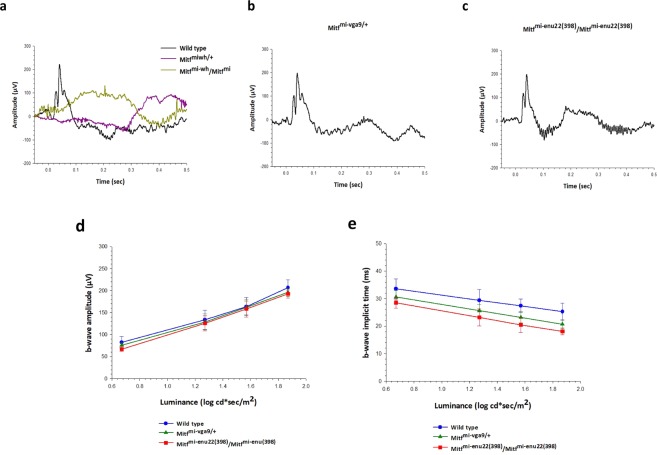
Light-adapted ERG recordings. (**a–c**) Representative ERG recordings from a wild type mouse (**a**, dark trace), and from mice carrying four different *Mitf* mutations in response to a single flash light stimulus of 1.87 log cd s/m^2^. (**d**,**e**) The relationship between the mean amplitudes of the light adapted (**d**) and the implicit time (**e**) ERG b-wave. One-way ANOVA followed by Bonferroni’s post-hoc comparisons test (wild type set as control) was used. Data are presented as means ± SEM, n = 6, P > 0.05.

Light-adapted ERG responses were recorded after 10 minutes of light adaptation with a steady white background light of 1.7 log cd/m^2^, which was kept present during the recordings, while evoking ERG responses with light flashes of increasing intensity. Figure 5a–c shows examples of photopic ERG responses recorded from wild type and *Mitf* mutant mice, evoked by the 1.87 log cd sec/m^2^ flash stimulus. As shown in Fig. 5a, the ERG recorded from normal mice showed a prominent b-wave whereas the *Mitf*^-*Mi*-*Wh*/+^and *Mitf*^*Mi*-*Wh*^/*Mitf*^*mi*^ mice showed a light-adapted ERG response that could not be detected clearly above baseline, suggesting complete lack of cone vision. In contrast, *Mitf*^*mi*-*vga9*/+^ and *Mitf*^*mi*-*enu22*(*398*)^/*Mitf*^*mi*-*enu22*(*398*)^ mice showed light-adapted ERG b-waves evoked by the 1.87 log cd sec/m^2^ flash stimulus that were comparable to the b-wave recorded from wild type mice. The mean amplitudes of the cone b-waves were not significantly different between those recorded from wild type mice (n = 6), *Mitf*^*mi*-*vga9*/+^ and *Mitf*^*mi*-*enu22*(*398*)^/*Mitf*^*mi*-*enu22*(*398*)^ mice (n = 6) at any of the levels of stimulus luminance tested (P>0.05) (Fig. 5d). The amplitude of the light-adapted b-wave increased in a comparable manner with increasing stimulus luminance in wild type (n = 6), *Mitf*^*mi*-*vga9*/+^ (n = 6) and *Mitf*^*mi*-*enu22*(*398*)^/*Mitf*^*mi*-*enu22*(*398*)^ mice (n = 6). The mean amplitude of the dark-adapted b-wave of the ERG in response to the same stimulus in wild type mice was 206.9 ± 17.7 µV, while it was 196.0 ± 9.2 µV in *Mitf*^*mi*-*vga9*/+^ mice, and 192.3 ± 8.994 µV in *Mitf*^*mi*-*enu22*(*398*)^/*Mitf*^*mi*-*enu22*(*398*)^ mice. No significant differences were observed in the b-wave implicit time in those mutants at any of the levels of stimulus luminance tested (P>0.05) (Fig. 5e). The mean amplitude of the b-wave implicit time in response to the 1.87 log cd sec/m^2^ flash stimulus in normal mice was 25.3 ± 3.1 µV, 20.7 ± 1.6 µV in *Mitf*^*mi*-*vga9*/+^ mice, and 18.1 ± 1.2 µV in *Mitf*^*mi*-*enu22*(*398*)^/*Mitf*^*mi*-*enu22*(*398*)^ mice.

### *Mitf*^*Mi*-*Wh*/+^ and *Mitf*^*Mi*-*Wh*^/*Mitf*^*mi*^ mutant mice show retinal degeneration

**Figure 6:**
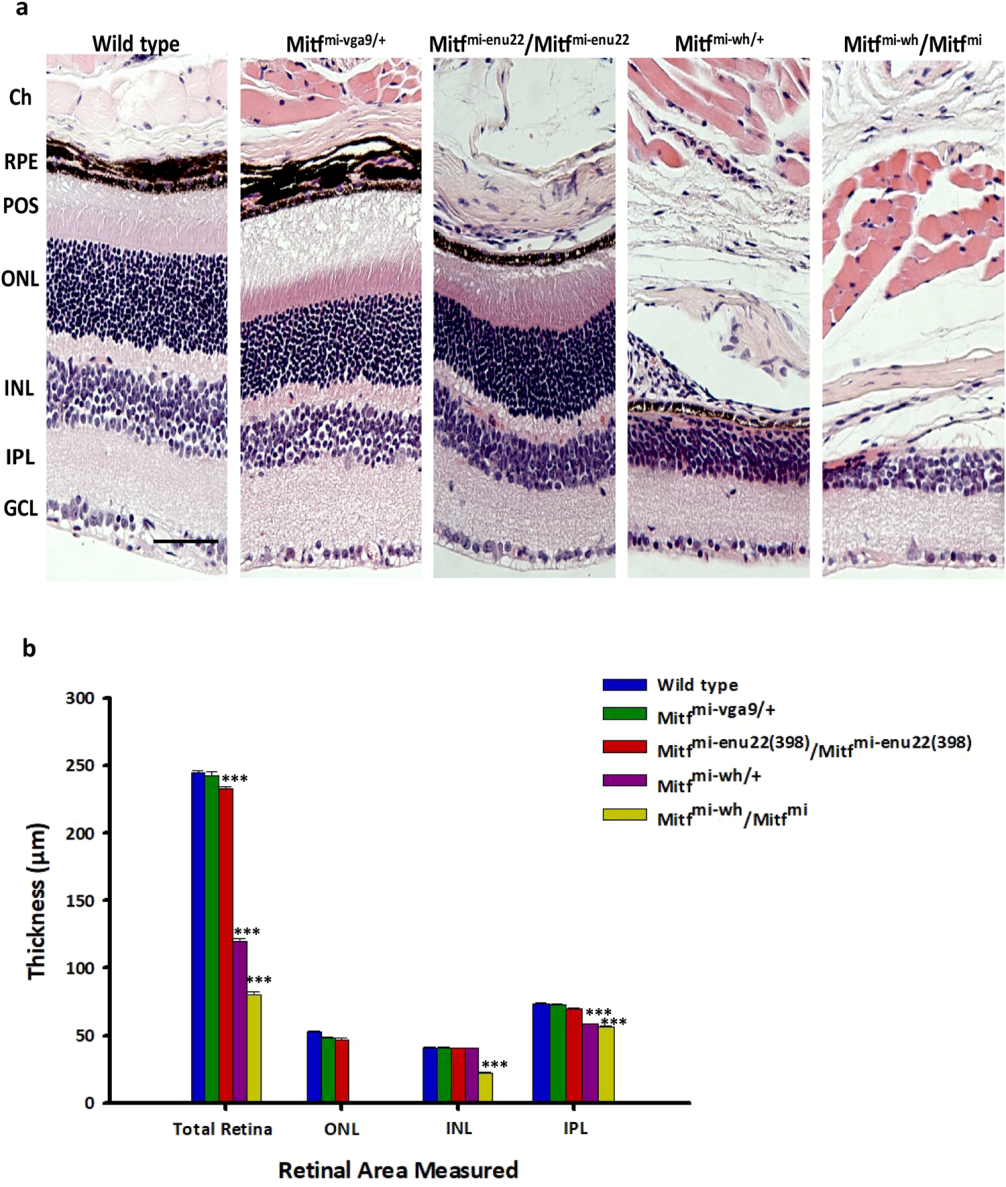
Histology and analysis of the retinal layers of eyes from wild type and *Mitf* mutant mice. Representative retinal sections of 3 months old mice are shown. (**a**) Central retina from wild type and mutant mice. (**b**) Analysis of the retinal layers. Total retina thickness from GCL to POS, ONL, INL and IPL thicknesses were measured from the sections. Scale bar: 50 µm. Ch, choroid; RPE, retinal pigment epithelium; POS, photoreceptor outer segments; ONL, outer nuclear layer; INL, inner nuclear layer; IPL, inner plexiform layer; GCL, ganglion cell layer. One-way ANOVA followed by Bonferroni’s post-hoc comparisons test (wild type set as control) was used. Data are presented as means ± SEM, n = 6, ***p < 0.001.

In order to assess the structures of mutant retinas at 3 months of age we examined tissue sections stained with haematoxylin and eosin (H&E). In eyes from *Mitf*^*mi*-*vga9*/+^ (n = 6) and *Mitf*^*mi*-*enu22*(*398*)^/*Mitf*^*mi*-*enu22*(*398*)^ (n = 6) animals all the layers of the retina and RPE were found to be present (Fig. 6a) although the total retina was significantly thinner in *Mitf*^*mi*-*enu22*(*398*)^/*Mitf*^*mi*-*enu22*(*398*)^ mutant mice than in wild type (P < 0.001) (Fig. 6b). The RPE is present in eyes from both *Mitf*^*mi*-*vga9*/+^ and *Mitf*^*mi*-*enu22*(*398*)^/*Mitf*^*mi*-*enu22*(*398*)^ animals, while the choroidal melanocytes adjacent to Bruch’s membrane are present in *Mitf*^*mi*-*vga9*/+^ eyes, but absent from the *Mitf*^*mi*-*enu22*(*398*)^/*Mitf*^*mi*-*enu22*(*398*)^ mutant mice (Fig. 5a). No significant differences were observed in the thickness of the outer nuclear layer (ONL) between *Mitf*^*mi*-*vga9*/+^ (n = 6) and *Mitf*^*mi*-*enu22*(*398*)^/*Mitf*^*mi*-*enu22*(*398*)^ (n = 6) compared to wild type mice (n = 6). However, *Mitf*^-*Mi*-*Wh*/+^and *Mitf*^*Mi*-*Wh*^/*Mitf*^*mi*^ mice showed clear evidence of retinal degeneration; we observed that photoreceptor outer segments (POS) and ONL were absent from animals of both genotypes (Fig. 6a), and the total retina and inner plexiform layer (IPL) were significantly thinner compared to wild type (P < 0.001). In addition, eyes from *Mitf*^*Mi*-*Wh*^/*Mitf*^*mi*^ animals showed thinner inner nuclear layer (INL) than wild type mice (Fig. 6b). The eyes of the *Mitf*^*Mi*-*Wh*^/*Mitf*^*mi*^ animals show no evidence of the RPE, Bruch’s membrane or choroidal melanocytes in histological sections, while a thin layer of RPE cells is present in sections of eyes from *Mitf*^-*Mi*-*Wh*/+^mice (Fig. 6a).

## Discussion

The different mutations at the mouse *Mitf* locus have different effects on the phenotype, ranging from no visible effects to deaf animals lacking melanocytes with severe microphthalmia and osteopetrosis (reviewed in^32^). Here we study eye function and morphology in mice carrying alleles which result in mild effects on adult eye size and apparent external structures; all have normal eye size. Our analysis shows that *Mitf* is not only important for normal adult eye size and external structure but is also critical for normal retinal function at 3 months of age. We showed that although eye size is normal in these mutants, pigmentation of the fundus is altered in all the mutants. The alterations in the fundus vary from a widespread lack of pigmentation to various degrees of hypopigmentation to yellow spots with non-pigmented areas (Table 2). We found that despite some depigmentation of the RPE, two of the mutants, *Mitf*^*mi*-*vga9*/+^ and *Mitf*^*mi*-*enu22*(*398*)^/*Mitf*^*mi*-*enu22*(*398*)^ not only have normal adult eye size and external structure but also have functioning retinas. The histology shown in Fig. 6a shows pigment in the RPE layer of these two mutants, although reduced, while choroidal melanocytes are near absent from *Mitf*^*mi*-*enu22*(*398*)^/*Mitf*^*mi*-*enu22*(*398*)^ mice but present in *Mitf*^*mi*-*vga9*/+^ mice. The pigmentation observed by fundus photography is dependent on the presence of melanin in both RPE cells and choroidal melanocytes, both of which are affected by *Mitf* mutations. Hypopigmentation observed in the fundus of these mice is not necessarily an indication of RPE dysfunction or retinal degeneration. However, it is of course possible that despite lack of morphological changes, these mutations affect the expression of important downstream target genes affecting photoreceptor function or integrity at older ages. For example, homozygous *Mitf*^*mi*-*vga9*^mice develop photoreceptor degeneration as they age due to effects on PEDF expression^48^. The other two mutants studied, *Mitf*^*Mi*-*Wh*/+^ and *Mitf*^*Mi*-*Wh*^/*Mitf*^*mi*^ have clear histological defects in the RPE layer and a near absence of choroidal melanocytes and show clear evidence of severe retinal degeneration at 3 months of age (summarized in Table 2**)**. Thus it appears that *Mitf* mutant mice with intact RPE layers have normal retinal function and structure at 3 months of age, even though there is a severe reduction of choroidal melanocytes. We have yet to determine the exact time course of these retinal degenerations, which may be either progressive and rapid, or present at birth and thus directly due to the effects of *Mitf* during eye development.

**Table 2:** Eye phenotype summary of the mutants. RPE, retinal pigment epithelium; POS, photoreceptor outer segments; ONL, outer nuclear layer.

Mutant mice	Fundus photography	Fluorecein angiography	Electroretinogram	Histology
*Mitf* ^*mi*- *vga9*/+^	Yellow lesions and minor hypopigmentation	Normal vasculature	Normal	All retinal layers are present
*Mitf*^*mi*-*enu22*(*398*)^/*Mitf*^*mi*-*enu22*(*398*)^	Large white lesions	Hyperfluorescent areas but with capillaries present	Normal	All retinal layers are present
*Mitf* ^*Mi*- *Wh*/+^	Hypopigmentation and comparable large non-pigmented areas	Hyperfluorescent regions across the entire fundus, and a clear reduction in the capillary network	Not above baseline	RPE, POS and ONL are absent
*Mitf*^*Mi*-*Wh*^/*Mitf*^*mi*^	Widespread lack of pigmentation and larger RPE lesions	No fluorescence in their retinal vasculature	Not above baseline	RPE, POS and ONL are absent

The electroretinographic findings indicate that mice carrying the *Mitf*^*mi*-*vga9*/+^ and *Mitf*^*mi*-*enu22*(*398*)^/*Mitf*^*mi*-*enu22*(*398*) ^mutations have normal dark-adapted and light-adapted ERG responses, while the other two, *Mitf*^-*Mi*-W*h*/+ ^and *Mitf*^*Mi*-W*h*^/*Mitf*^*mi*^ mice, show no ERG responses to any stimuli under either condition (Table 2). The compound heterozygote *Mitf*^*Mi*-*Wh*^/*Mitf*^*mi*^ carries two different alleles in the *Mitf* gene. The semidominant *Mitf*^*Mi*-*Wh*^ mutation results in grey coat in heterozygotes and white coat in homozygotes; eye development is normal in the heterozygote whereas intermediate microphthalmia is seen in the homozygote (Table 1)^32,47^. In the homozygous condition, the *Mitf*^*mi*^ mutation leads to white, microphthalmic mice which die at 3 weeks of age due to osteopetrosis; heterozygotes are normal apart from a small belly spot^23,31^. The *Mitf*^*Mi*-*Wh*/^*Mitf*^*mi*^ compound heterozygote is of particular interest since it shows interallelic complementation as its phenotype is more normal with respect to eye development than the phenotype of each homozygote alone^31^. Interestingly, we observed that *Mitf*^*Mi*-*Wh*/+^ and *Mitf*^*Mi*-*Wh*/^*Mitf*^*mi*^ mice have no ERG response and the retina is abnormal at 3 months of age. The normal development of a full size eye, as induced by the interallelic complementation, is clearly not sufficient to prevent severe and early degeneration. Our results show that the *Mitf*^*Mi*-*Wh*/+^ mutant is clearly semidominant with respect to eye function. Interestingly, these mice show a profound hearing dysfunction due to an absence of melanocytes from the post natal cochlea^34^ suggesting semidominant action also in the ear. The hearing abnormality present in these mice reinforces the notion of their similarity to individuals with Waardenburg and Tietz syndromes. Waardenburg syndrome, of which there are four subtypes with different penetrance of the clinical features, is a congenital, dominantly inherited pigmentation anomaly characterized by deafness, hypopigmentation of the skin, hair and eyes, with a midline white forelock of hair, and in some cases facial anomalities^37,49,50^. Ocular symptoms include iridial heterochromia, hypopigmentation of the fundi^36,50–52^, and in rare cases unilateral macular degeneration have also been observed^53^. Tietz syndrome patients show more severe symptoms of the same kind, and they are invariably deaf while approximately 75% of Waardenburg syndrome patients are deaf or have a severe hearing loss^38,39^. This shows again, similarities in human and mouse phenotypes, although reports on visual function or electroretinography performed on Waardenburg or Tietz patients are scant or in most cases non-existent.

In previous electroretinographic studies of other mice with mutations in the *Mitf* gene, the results have varied. Some of the mutations, such as *Mitf*^*mi*-*bws*^/*Mitf*^*mi*-*bws*^ and *Mitf*^*mi*-*sp*^/*Mitf*^*mi*-*sp*^ mice have normal ERGs, or in the case of *Mitf* ^*Mi*-*Wh*^/*Mitf* ^*mi*-*sp*^ mice, a reduction in all components of the response^23^. A slow, progressive loss of the ERG b-wave occurs in the homozygous *Mitf*^*mi-vit*^/*Mitf*^*mi-vit*^ mice^29,54^. However, homozygous *Mitf*^*Mi*-*Wh*^/*Mitf*^*Mi*-*Wh*^ mice show no ERG response at 3 months of age^23^, as found in two of the mutant mice in the present study. These results taken together suggest that even with changes in the pigmentation and morphology of the RPE caused by specific mutations in the *Mitf* gene, such as in *Mitf*^*mi*-*vga9*/+^ and *Mitf*^*mi*-*enu22*(*398*)^/*Mitf*^*mi*-*enu22*(*398*)^ mice, the photoreceptors and the inner layers of the neuroretina may survive and function normally.

Light microscopy analysis of histological sections of the eyes of the mutants revealed that the RPE layer in the *Mitf*^*mi*-*enu22*(*398*)^/*Mitf*^*mi*-*enu22*(*398*)^ mice shows localized thinning of melanin-containing areas. This is probably due to the localized degeneration of choroidal melanocytes in these animals. An equivalent light microscopy analysis of the eyes of *Mitf*^*mi*-*vga9*/+^ mice shows a normal RPE layer and apparent choroidal melanocytes, although fundus images reveal fine pigment mottling as white spots across the fundus. The *Mitf*^-*Mi*-*Wh*/+^ mice showed a near complete absence of the melanin-containing areas of the RPE layer, and *Mitf*^*Mi*-*Wh*^/*Mitf*^*mi*^ mice showed a thin monolayer of RPE cells but absence of apparent choroidal melanocytes or a Bruch’s membrane. Retinal thickness analysis showed that the POS and ONL layers are absent from *Mitf*^-*Mi*-*Wh*/+^ and *Mitf*^*Mi*-*Wh*^/*Mitf*^*mi*^ eyes; the IPL is thinner compared to wild type. In addition, the *Mitf*^*Mi*-W*h*^/*Mitf*^*mi*^ mice showed thin INL compared to normal mice. In both cases, the major phenotypic effects can be attributed to the *Mitf*^-*Mi*-*Wh*^ allele which clearly has severe effects on the eye. Despite substantial thinning of the melanin-containing areas of the RPE layer in *Mitf*^*mi*-*enu22*(*398*)^/*Mitf*^*mi*-*enu22*(*398*)^ mice, due to absence of choroidal melanocytes, and some hypopigmentation and pigment mottling in the fundi of *Mitf*^*mi*-*vga9*/+^ mice, no significant differences were observed in the thickness of the ONL, INL and IPL in these mice as compared to wild type mice; the total retina is thinner in the *Mitf*^*mi*-*enu22*(*398*)^/*Mitf*^*mi*-*enu22*(*398*)^ mice than in either wild type or *Mitf*^*mi*-*vga9*/+^ mice.

This study provides more evidence that a functional RPE is important for normal photoreceptor function. Thus, mutations in genes expressed in the RPE such as *Mitf* and *Otx2*, can have profound effects on retinal structure and function, as a consequence of alterations in the RPE^17,18,55^. RPE-specific ablation of *Otx2* in the adult mouse retina leads to RPE dysfunction, which in turn leads to photoreceptor degeneration comparable to those that occur in *Mitf*^*Mi*-*Wh*/+^ and *Mitf*^*Mi*-*Wh*^/*Mitf*^*mi*^ mice^17,18^. Mutations in the human *OTX2* gene on chromosome 14q22.3, which codes for a transcription factor that shares 100% amino acid identity with its mouse ortholog (*Otx2*), cause pattern dystrophy of the RPE, and slow photoreceptor degeneration in patients^19^, similar to what occurs in mice with *Otx2* ablation^17,18^. However, unlike *Mitf*, *Otx2* is also expressed in the bipolar and photoreceptor cells of the retina, in addition to its role in regulating visual cycle function in the RPE^17,20^. Thus, it can be stated with certainty that retinal dysfunction or degeneration in *Mitf* mutant mice found here are due to mutations in a gene which in the eye is only expressed in the RPE^28^, while those seen in *Otx2* mutant mice, and in patients with *OTX2* mutations, are more complex.

The RPE plays a vital role in the visual cycle^56^. This process is a key element of the interactions between the RPE cells and photoreceptor outer segments. In the RPE, *Mitf* is known to regulate the expression of two visual cycle genes, *Rlbp1* which encodes retinaldehyde binding protein-1 (RLBP1), and *Rdh5*, which encodes retinol dehydrogenase-5 (RDH5)^55^. Thus, *Mitf* controls expression of visual cycle genes and consequently the reconstitution of functional rhodopsin, although not the same processes of the visual cycle as *Otx2* in the RPE. Wen *et al*.^55^ also found that postnatal injection of 9-cis-retinal can partially rescue the retina in homozygous *Mitf*^*mi*-*vga9*^ mutant mice with respect to gene expression, structure and function. This means that the lack of *Mitf* in the RPE may have such profound developmental effects that the adjacent retina might be permanently damaged^55^. It is likely that these processes of the visual cycle play a role in inducing the degeneration found in the eyes of *Mitf*^*Mi*-*Wh*/+^ and *Mitf*^*Mi*-*Wh*^/*Mitf*^*mi*^ mice. We have shown here that mutations in a transcription factor expressed in the RPE, and not in the neuroretina, have profound effects on the appearance of the fundus of the eye, in particular its pigmentation, and can lead to changes in neuroretinal function, and to severe retinal degeneration in some cases. The study further demonstrates that the RPE is essential for vision, and that transcription factors whose expression in the eye is restricted to the RPE play a role that is critical for normal retinal structure and function.

## Material and Methods

### Animals

The following 3-month-old *Mitf* mutants were used in this study: C57BL/6J-*Mitf*^*mi*-*vga9*/+^, C57BL/6J-*Mitf*^*mi*-*enu22*(*398*)^/*Mitf*^*mi*-*enu22*(*398*)^, C57BL/6J-*Mitf*^*Mi*-*Wh*/+^, and C57BL/6J-*Mitf*^*Mi*-*Wh*^/*Mitf*^*mi*^ (Table 1). The mice were kept at the University of Iceland. All animal experiments were approved by The Icelandic Food and Veterinary Authority (MAST licence numbers 2017-04-03 and 2018-05-02) and were performed in accordance with the Association for Research in Vision and Ophthalmology Statement for the Use of Animals in Ophthalmic and Visual Research.

### Fundus photography

Live fundus photographs were obtained from anaesthetized mice with a Micron IV rodent fundus imaging system (Phoenix Research Labs Inc., Pleasanton, CA). Prior to fundus examination the mouse was given an intraperitoneal injection of a mixture of 40 mg/kg^−1^ Ketamine (Pfizer, Denmark) and 4 mg/kg^−1^ Xylazine (Chanelle Pharmaceuticals, Ireland), and the anaesthesia was maintained as needed with half of that dose. Pupils were dilated with topical application of Mydriacyl (1% tropicamide) (Alcon Inc., U.S.A) eyedrops, and Alcaine (Alcon, Inc., U.S.A) drops were applied as a local corneal anaesthetic. A photograph of the front of the anterior chamber of the eye was then taken with the fundus camera to assess both eye size and pupil dilation. A 1% methylcellulose gel was applied to the cornea of both eyes and a mini contact lens (Ocuscience LLC, Henderson, NV) 2.5 mm in diameter placed on the cornea, to reduce the risk of cataract formation^57^. The contact lens from one eye was removed before fundus photography and 1% methylcellulose applied to the eye again. A bright field image of the anterior segment was first obtained, and then bright field images of the fundus with the optic nerve in the centre of the field. Fluorescent angiography fundus images were obtained after intraperitoneal injection of fluorescein sodium salt (0.1%, 10 µl/kg).

### Electroretinogram (ERG)

#### Instrumentation

The electroretinogram (ERG) was recorded between two electrodes with a MacLab ML131 BioAmp amplifier. A coiled urethane coated stainless steel wire (0.2 mm in diameter) was used as the corneal ERG electrode^58^. One platinum wire was placed in the chin of the mouse for reference^59^ and another one in the outer ear, serving as ground. The signal was amplified 1000 times. The recording was fed into a computer through a PowerLab analogue/digital converter (ADInstruments Pty Ltd., NSW, Australia). Data acquisition and analysis was performed with the LabChart 7 Pro software package (ADInstruments Pty Ltd., NSW, Australia). Voltage responses were amplified 10.000 times and passed through a 50 Hz notch filter and band pass filters set at 0.3–500 Hz. In the LabChart Pro software a 100–500 Hz digital band pass filter was set to isolate the oscillatory potentials (OPs) in a separate recording channel. The sampling rate of the recordings was set at 20 kHz. White light stimuli, with a duration of 10 µs to evoke the ERG, were generated with a Grass PS-33 photostimulator (Astro-Med/Grass Inc., West Warwick, RI, USA), and presented with a Ganzfeld strobe. The light stimuli used were of four different luminances: 0.67, 1.27, 1.57 and 1.87 log cd sec/m^2^.

#### Recording procedure

Prior to each ERG experiment the mouse was given an intraperitoneal injection of 40 mg/kg^−1^ Ketamine and 4 mg/kg^−1^ Xylazine and then the anaesthesia was maintained with half of that dose. The electrode always contacted the cornea through a layer of 1% methylcellulose^58^. The pupil was dilated with topical application of Mydriacyl (1% tropicamide) (Alcon Inc., U.S.A) and Alcaine (Alcon, Inc., U.S.A) drops were used as a local corneal anesthetic. Saline solution drops were systematically applied to the eye as a tear supplementation, in order to prevent dehydration and cataract formation, and to provide adequate electrical conduction for the corneal electrode. In order to further prevent cataract formation, a mini contact lens (Ocuscience LLC, USA) completely covered the cornea through a layer of 1% methyl cellulose gel during dark adaptation^57^, which was removed under dim red illumination before placing the corneal electrode. The mouse was placed on a heating pad with a rectal thermal sensor beneath it, both connected to a thermal regulator. The head of the mouse was positioned in a straight line with the body, by a short string threaded behind its upper front teeth, to maintain normal breathing. The mice were dark adapted for 30 minutes before recording the scotopic ERG responses. At least one minute elapsed before stimulus presentations in order to allow the photoreceptors to recover from any photopigment bleaching and to maintain the dark adaptation state between flashes^59^. Afterwards the mouse was light adapted with a steady background light of 1.7 log cd/m^2^ for ten minutes. The ERG recording protocol was the same after light adaptation as the one following dark adaptation. At the end of the experiment the anaesthetized animal was sacrificed via cervical dislocation and then bled by femoral artery sectioning.

#### ERG data analysis

The amplitudes of the ERG and its components are presented as means ± standard error of the mean, in microvolts (µV). The a-wave amplitude was measured from baseline to the lowest base of the cornea-negative deflection. The b-wave was either measured from baseline or lowest point of the a-wave (if present), to its peak. Implicit times of the a-, b-waves were measured from flash onset and until their apex^59,60^. Oscillatory potentials (OPs) were extracted with a band-pass filter (100–500 Hz). The peaks of the first four OPs were measured and summed to generate the total OP amplitude as previously described^61,62^. The amplitude of each OP was measured individually from the preceding trough to the peak of the OP considered, except for OP1 that was measured from the preceding baseline to its peak.

### Histology and measurement of the retinal layers

Mice were euthanized by cervical dislocation. Eyes were enucleated and fixed in 4% paraformaldehyde over night at 4 °C. Subsequently, the eyes were embedded in paraffin and sectioned at 5 µm onto glass slides. Retinal tissue sections were deparaffinised and then stained with hematoxylin and eosin (H&E). Microscopic examination was performed using an Olympus IX71 microscope (Tokyo, Japan). The distance from the retinal surface to the end of the photoreceptor outer segments was measured as the retinal thickness. The thicknesses of the ONL, INL and IPL in the central retina were measured by light microscopy at the point where the ratio of the distance from the optic disc to the point to the distance from the optic disc to peripheral end of the retina was 0.2^63^. The values recorded for each parameter for the nasal and temporal sides were averaged to obtain a single value for each eye. For consistency, only retinal sections with optic nerve stumps were used.

### Statistical analysis

All data are presented as mean ± SEM. One-way ANOVA followed by Bonferroni’s post-hoc comparisons test was performed in all statistical analyses. Values of P > 0.001 were considered statistically significant (indicated with ***). Statistical analysis and graphs were plotted using SigmaPlot 13 (Systat Software, San Jose, CA).

## Data Availability

The datasets generated during and/or analysed during the current study are available from the corresponding author on reasonable request.
